# Preparation of Plasmonic Ag@PS Composite *via* Seed-Mediated *In Situ* Growth Method and Application in SERS

**DOI:** 10.3389/fchem.2022.847203

**Published:** 2022-03-11

**Authors:** Xiaoran Tian, Qian Yu, Xianming Kong, Miao Zhang

**Affiliations:** ^1^ School of Petrochemical Engineering, Liaoning Petrochemical University, Fushun, China; ^2^ Department of Materials and Environmental Chemistry, Stockholm University, Stockholm, Sweden

**Keywords:** seed-mediated *in situ* growth, SERS, on-site, sensing, Ag@PS

## Abstract

The colloidal polystyrene (PS) was synthesized and decorated with silver nanoparticles (Ag NPs). The plasmonic Ag@PS nanocomposite was prepared by loading Ag NPs on PS microsphere through a seed-mediated *in situ* growth route. The property of Ag NPs deposited on the PS microsphere could be precisely controlled by adjusting the concentration of the chemicals used in the growth medium. The growth step is only limited by the diffusion of growing species in the growth media to the surface of the Ag seed. The Ag@PS prepared via the *in situ* growth method exhibited two advantages compared with the self-assembled PS/Ag. First, the high-density of Ag NPs were successfully deposited on the surface of PS as the electroless-deposited Ag seed process, which brings nearly three times SERS enhancement. Second, the rapid preparation process for *in situ* growth method (half an hour, 10 h for the self-assembled method). The PS/Ag could detect Nile blue A (NBA) down to 10^–7^ M by SERS. Furthermore, the plasmonic Ag@PS SERS substrate was used for pesticide identification. The on-site monitoring malachite green (MG) from fish was achieved by portable Raman spectrometer, and the limit of detection (LOD) was 0.02 ppm. The Ag@PS substrate has also shown capability for simultaneously sensing multiple pesticides by SERS.

## Introduction

Surface-enhanced Raman scattering (SERS) spectroscopy has several advantages, such as high sensitivity, rapid detection speed, specificity, and nondestructiveness, which has become one of the essential analytical techniques (Cao et al., 2002; Kneipp et al., 2008). SERS has been commonly applied in chemical reaction monitoring, surface science, medical diagnosis, food testing, and biochemistry (Zhang et al., 2015; Haruna et al., 2016; Kamińska et al., 2016; Yuan et al., 2017; Kong et al., 2018). The SERS phenomenon was first discovered in 1970s by Fleichman and his colleagues (Fleischmann et al., 1974). The metallic NPs were usually applied in SERS with the emergence of nanoscience, which extremely expanded the scope of application of SERS. The nanomaterials of noble metal, especially silver and gold are widely used to construct SERS substrates (Onawole et al., 2018; Lin et al., 2020; Sivashanmugan et al., 2019a). The preparation of active substrate is critical for the application of SERS (Tao et al., 2003; Robinson et al., 2015; Sivashanmugan et al., 2019b). The development of simple and effective enhanced substrate has become one of the most significant challenges for SERS technology.

In recent years, the plasmonic composite was developed and used in SERS sensing because the composite could enable additional enhancement or new function to the SERS substrate (Sihan Zhang et al., 2021). The composite SERS substrates mainly include silicon, quartz, glass, copper, fibers, paper, swabs, eggshells, graphene oxide, and polymers (Li et al., 2013; Tao et al., 2013; Gong et al., 2014; Kim and Min, 2014; Hou et al., 2015; Hu et al., 2015; Kong et al., 2015; Jin et al., 2016; Yaling et al., 2017; Zhang et al., 2019). These composite materials composed of polymer and metallic NPs have attracted great interest, as the composite has shown multiple functions (Cai et al., 2013). The composite SERS substrate was fabricated by decorating polymer microspheres with metallic NPs. These composites were widely used in the fields of photonic crystals (Gittins et al., 2002), plasmon resonance (Xiao et al., 2010), and SERS (Capek et al., 2005). PS microsphere was combined with metallic NPs to prepare SERS substrates due to the strong adsorption capacity, strong oxygen permeability, and the possibility of surface functionalization. Several methods have been developed to fabricate metal/PS composites, for example, self-assembly procedures, surface reduction reactions, and magnetron sputtering (Wei et al., 2007; Ishida et al., 2008; Cao et al., 2009; Chang et al., 2010; Kim et al., 2010; Jian et al., 2013; Balčytis et al., 2017). Ocwieja et al. (2018) produced AuNP-PS nanoparticles/particles by forming a layer of positively charged gold nanoparticles on polystyrene. Lee et al. (2010) prepared PS@Au by loading Au NPs onto the surface of sulfonated PS, in which the *in situ* ion exchange method was used for Au depositing. Li et al. (2014) fabricated PS/Ag nanocomposite and used as enhanced substrate to detect pesticides; the detection limit for organophosphorus was down to 96 nM. The current methods are mainly focused on self-assembly method that was time consuming, or a special instrument is needed. Furthermore, it was difficult to deposit dense metallic NPs on the surface of PS through self-assembly processes with the limited diffusion rate of the metallic colloid.

MG is commonly applied as a dye in the silk, leather, and paper industries. It is also used as an insecticide or fungicide in the aquaculture industry (Forgacs et al., 2004; He et al., 2008). The carcinogenic and genotoxic properties of MG would pose a severe threat to human health (Stead et al., 2010; Sivashanmugan et al., 2015; Yuanyi Zhang et al., 2021). Many countries have banned the application of MG in aquatic products (Xu et al., 2019). Therefore, a simple and instant technique is necessary for monitoring trace level of MG from aquaculture products. There are several techniques that have been used to identify MG, such as liquid chromatography-tandem mass spectrometry (LC-MS) (Halme et al., 2007), enzyme-linked immunosorbent assay (ELISA) (Yang et al., 2007), flow injection analysis (FIA) (Heras and Pollero, 1990), capillary electrophoresis (Bergwerff and Scherpenisse, 2003), and biochip technology (Marquette et al., 2006). However, most of these technologies were expensive and complicated.

In this paper, PS colloid was synthesized through emulsifier-free emulsion polymerization process, and then Ag@PS composites were prepared by *in situ* growth. Dense Ag NPs were immobilized on the surface of solid support because the homogeneous growth process could overcome the limitations of mass diffusion. In the preparation process, the Ag seeds were first loaded on the surface of PS microsphere *via* electroless deposition. After that, the Ag seeds grew to Ag NPs with a bigger diameter by immersing in growth medium. The preparation process was within 10 min, and the Ag@PS composite showed excellent SERS activity, which was also successfully applied for sensing MG from the surface of fish.

## Experiment

### Chemicals

Ascorbic acid (AA, 99%), hydrochloric acid (HCl), and Tin (II) chloride dihydrate (SnCl_2_, 98%) were obtained from Sinopharm Chemical Reagent Co., Ltd. (Shanghai, China). Silver nitrate (AgNO_3_, 99.7%), NBA, MG, P-aminothiopheno (PATP, 98%), styrene (C_8_H_8_), and 4-mercaptobenzoic acid (4-MBA, 90%) were obtained from Aladdin (Shanghai, China). Sodium 4-vinylbenzenesulfonate (C_8_H_7_SO_3_Na, 90%), potassium bicarbonate (KHCO_3_, 99.5%), and potassium persulfate (K_2_S_2_O_8_, 99%) were purchased from Innochem (Beijing, China). All chemicals were directly used without purification.

### Synthesis of Polystyrene Microspheres

C_8_H_8_ (13 ml), KHCO_3_ (0.50 g), C_8_H_7_SO_3_Na (0.0103 g), and water (100 ml) were mixed in a flask (250 ml) and heated gradually. When the solution was heated to 72°C, K_2_S_2_O_8_ (0.074 M 25 ml) was added into the system in 30 min. The reaction system was kept stirred for 8 h at 72°C and cooled to 25°C. The solid product was separated through centrifugation and cleaned with ethanol. The obtained PS powder was dispersed in ethanol.

### Preparation of Plasmonic Ag@PS Composite

The aqueous solution of SnCl_2_ and HCl (20 mM, 2 ml) was mixed with the PS (0.02 g/ml 1 ml) suspension for 3 min to deposit Sn^2+^ on the PS microspheres. After centrifugation and washing with water, the PS microspheres were redispersed into 400 μl of deionized water and mixed with 2 ml of AgNO_3_ (20 mM) solution for 3 min to deposit the Ag seeds. PS microspheres with Ag seeds were isolated by centrifuging and redispersing into the growth solution containing AgNO_3_ and AA for 3 min to form Ag NPs. The Ag@PS composite material was obtained by centrifugation.

### Surface-Enhanced Raman Scattering Measurement

Raman spectra was measured on a portable Raman spectrometer (BWS465 iRman; B&W Tek, United States). The wavelength of the laser was 785 nm, and the size of the laser spot was 105 µm. The laser power was 30% with a 2-s acquisition time. The raw data were processed by the BWSPEC software.

### Other Characterizations

The surface morphology of the nanocomposite was determined by an electron scanning microscope (SEM, SU8010, Hitachi, Japan) and transmission electron microscopy (TEM, JEM-2100FS JEOL, Japan). UV–vis absorption spectra of Ag@PS composite were obtained on a Cary 5000 spectrophotometer (Agilent, United States). Fourier transform infrared (FTIR) spectrum of the PS and Ag@PS composites was obtained from the Nicolet 6700 spectrometer (PerkinElmer, United States).

## Results and Discussion

**FIGURE 1:**
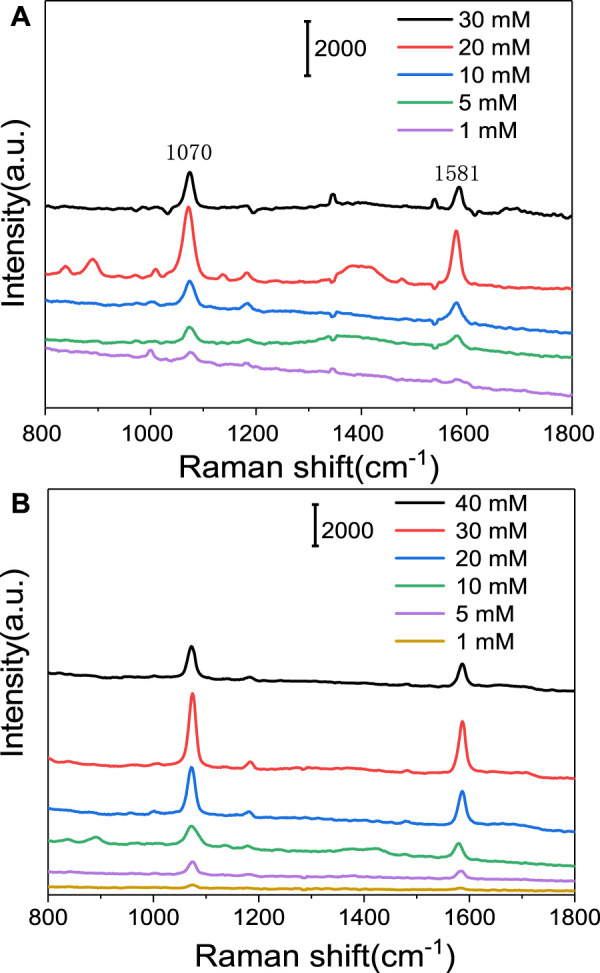
Raman spectra of 4-mercaptobenzoic acid (4-MBA) on Ag@PS composite that was *in situ* grown in growth media with different concentrations of silver nitrate (AgNO_3_) **(A)** and ascorbic acid (AA) **(B)**.

The SERS performance of Ag@PS was highly related to the density and size of the Ag NPs decorated on the surface PS microsphere. The AgNO_3_ and AA that existed in the growth medium were crucial to the property of Ag NPs. To prepare Ag@PS with the best SERS enhancement, the parameters in growth media were optimized. The various concentrations of AgNO_3_ and AA were used to prepare Ag@PS. The MBA was selected as a probe molecule in evaluating the SERS performance of the Ag@PS. Figure 1A presents the Raman spectra of 4-MBA from a composite, in which the Ag@PS were prepared with different concentrations of AgNO_3_ (AA, 20 mM). The characteristic Raman spectra of MBA were observed, and there were two prominent bands at 1,070 and 1,581 cm^−1^. The band at 1,070 cm^−1^ resulted from the stretching vibration of the C–S bond, and the band at 1,581 cm^−1^ was due to the breathing vibration of the aromatic ring. The SERS spectra were gradually increased as the concentration of AgNO_3_ varied from 1 to 20 mM. During the *in situ* growth process, Ag^+^ was reduced to Ag by AA, and the Ag seeds were deposited, which grew into Ag NPs with a bigger diameter. When a low concentration of AgNO_3_ presented in the growth media, the Ag NPs with a smaller diameter was formed due to the lack of Ag^+^ source. As the concentration of AgNO_3_ reached 20 mM. the Ag NPs with a bigger diameter and high density were formed on the surface of PS microsphere. The SERS enhancement effect was dependent on local electromagnetic fields via localized surface plasmon modes in plasmonic nanostructures. The Ag NPs with bigger diameter and high density could provide high SERS enhancement. While the 30 mM AgNO_3_ was used in the growth media, the intensity of the Raman spectra was decreased. The Ag NPs still grew and could form Ag shell on the PS microsphere as a high concentration of AgNO_3_ was used in the growth media. When the Ag NPs increased to very large particles, there was an increase not only in the electromagnetic field but also in the scattering efficiency, which resulted in weak Raman signals (Stamplecoskie et al., 2011). The concentration of AA was also optimized to obtain the best SERS activity, in which the various concentrations of AA were added in the growth media (AgNO_3_, 20 mM). The concentrations of AA were set from 1 to 40 mM. The SERS spectra of MBA prepared in various concentrations of AA are presented in Figure 1B, in which 30 mM of AA exhibited the best SERS enhancement. Further increasing the concentration of AA to 40 mM results in a decrease in the SERS activity because the Ag shell formed on the surface on the PS microsphere.

**FIGURE 2:**
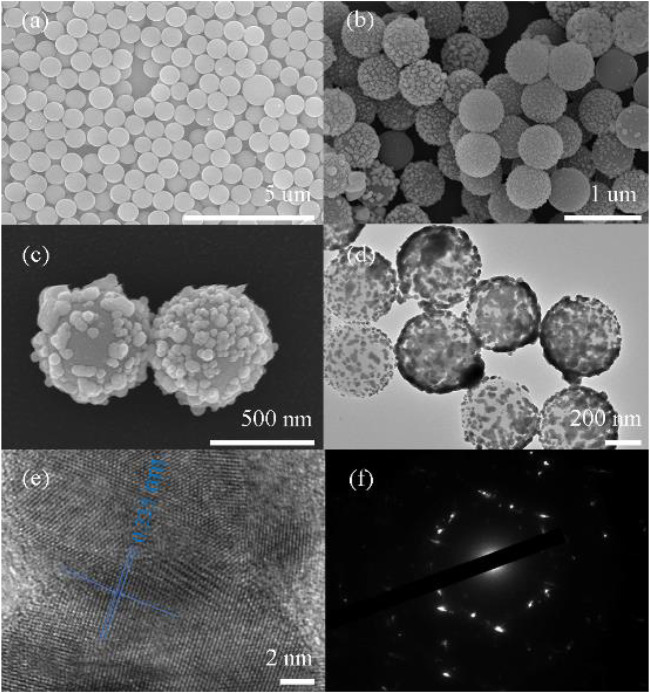
Scanning electron microscopy (SEM) images of polystyrene (PS) microspheres **(A)** and Ag@PS composites **(B,C)**. Transmission electron microscopy (TEM) image of the Ag@PS composite **(D)** and HRTEM image of Ag on PS **(E,F)**. SAED image of Ag NP.

The surface morphography of PS and Ag@PS was characterized by SEM and TEM images. Figure 2A is an SEM image of the PS microspheres; the PS microspheres show a smooth and clean surface, and the mean size is nearly 700 nm. The concentrations of AgNO_3_ and AA were set at 20 and 30 mM, respectively. After depositing the Ag NPs on PS through the *in situ* growth method, the Ag NPs were formed on the surface of PS as presented in Figure 2B. The surface roughness of Ag@PS was significantly increased compared with the PS microsphere. The diameter of the Ag NPs was distributed nearly from 40 to 60 nm as shown in Figure 2C. The elemental mapping of Ag is shown in Supplementary Figure S1, which indicated the successful decoration of Ag NPs on the surface of the plasmonic composite. The TEM image of the Ag@PS composite is presented in Figure 2D. The PS/Ag nanostructures were obviously observed as a strong contrast between the black Ag NPs and gray PS microspheres that further verified the deposition of Ag NPs on the surface of PS. Figure 2E presents the HRTEM image of the Ag NP on the PS. The randomly selected Ag NPs exhibited a face-centered cubic lattice (FCC), and the interlayer spacing of Ag (111) was 0.235 nm. Figure 2F was the SAED image of a single Ag NP. A series of diffraction rings were observed, which indicated that the Ag crystal was polycrystalline.

**FIGURE 3:**
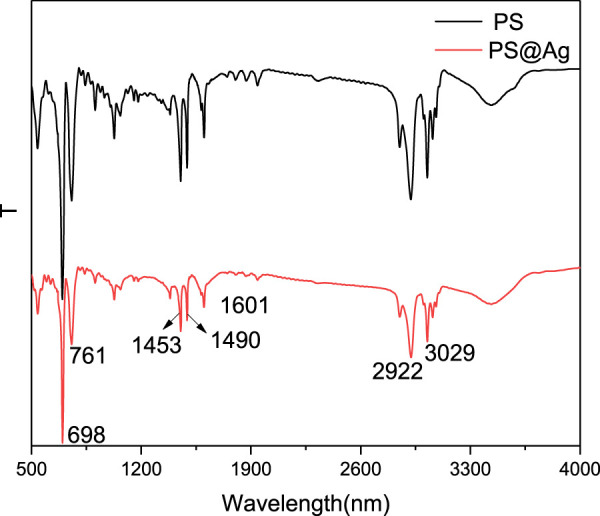
Fourier transform infrared (FTIR) of PS and Ag@PS composite.

The surface group of PS and Ag@PS were determined by FTIR spectrum as exhibited in Figure 3. The typical infrared bands of PS were observed at 698, 761, 1,453, 1,490, 1,601, 2,922, and 3,029 cm^−1^. The peak at 1,453 cm^−1^ was attributed to the symmetrical and asymmetrical angular deformation of CH_2_. The bands at 1,490 and 1,601 cm^−1^ were related to the stretching vibration of the C=C double bond in the aromatic ring. The peak at 2,922 cm^−1^ was due to the vibration of methylene groups. The peak at 3,029 cm^−1^ was attributed to the axial deformation of the aromatic C–H bond (El-Khiyami et al., 2021). The infrared spectrum of Ag@PS was similar with PS, but the intensity was weak. The reason was that the silver layer affects the interaction between infrared light and PS. The results indicated that the deposition of Ag NPs will not change the chemical properties of PS.

**FIGURE 4:**
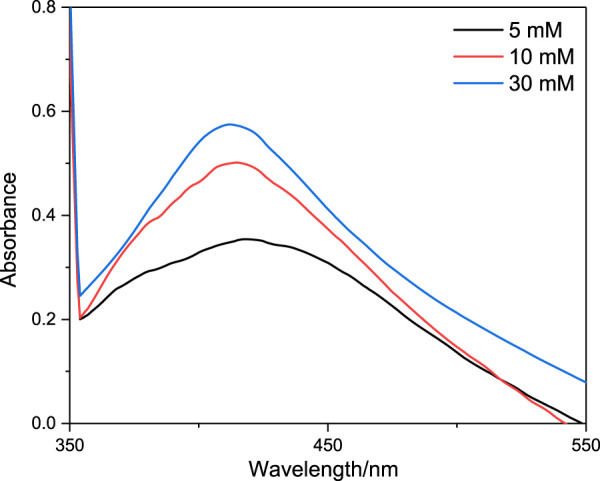
UV-Vis absorption spectra of Ag@PS composite synthesized with different concentrations of AA.

The UV-Vis spectra were employed to determine the plasmonic feature of Ag@PS composites. The Ag@PS were dispersed in water, and the UV-Vis spectra are presented in Figure 4, in which the Ag@PS were synthesized with various concentrations of AA. The absorbance peaks were presented nearly at 400 nm, which resulted from the LSPR of Ag NPs. The location and intensity of the absorbance band of Ag@PS composites are related with the size and density of the Ag NPs. As the 5 mM AA was used, the weak adsorption spectra were obtained. The intensity of LSPR peak was increased as the concentration of AA increased, which indicated that more Ag NPs were decorated on the surface of PS.

**FIGURE 5:**
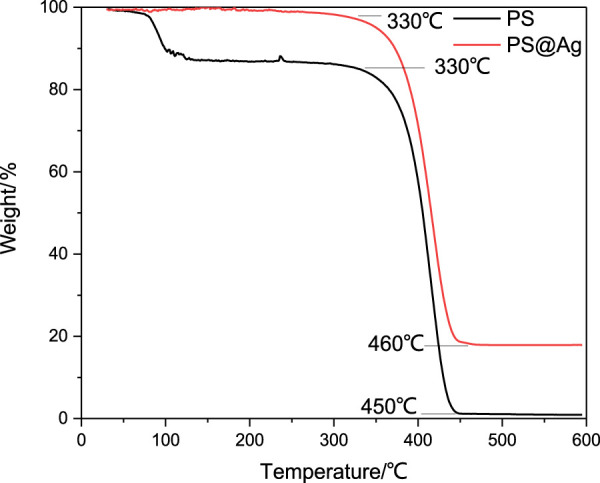
Thermogravimetry (TG) of PS microspheres and Ag@PS composite.

The thermal decomposition of the PS and Ag@PS composite were characterized through thermogravimetry as shown in Figure 5. For PS microspheres, there were two weight loss regions. The first region was nearly at 100°C, which was assigned to water included in the PS composites. The second weight loss region started at nearly 330°C, which was attributed to the decomposition of PS. The PS was almost decomposed completely when the temperature was higher than 450°C. The weight loss of Ag@PS composites started nearly at 330°C, that is, similar with the pure PS microsphere. The decomposition of Ag@PS was finished at nearly 450°C, and there was no weight loss that happened at higher than 450°C. The weight residue of Ag@PS was nearly at 17 wt%, which was due to the Ag NPs decorated on the PS. The difference in thermal decomposition between PS and Ag@PS indicates the high density of Ag NPs deposited on PS.

**FIGURE 6:**
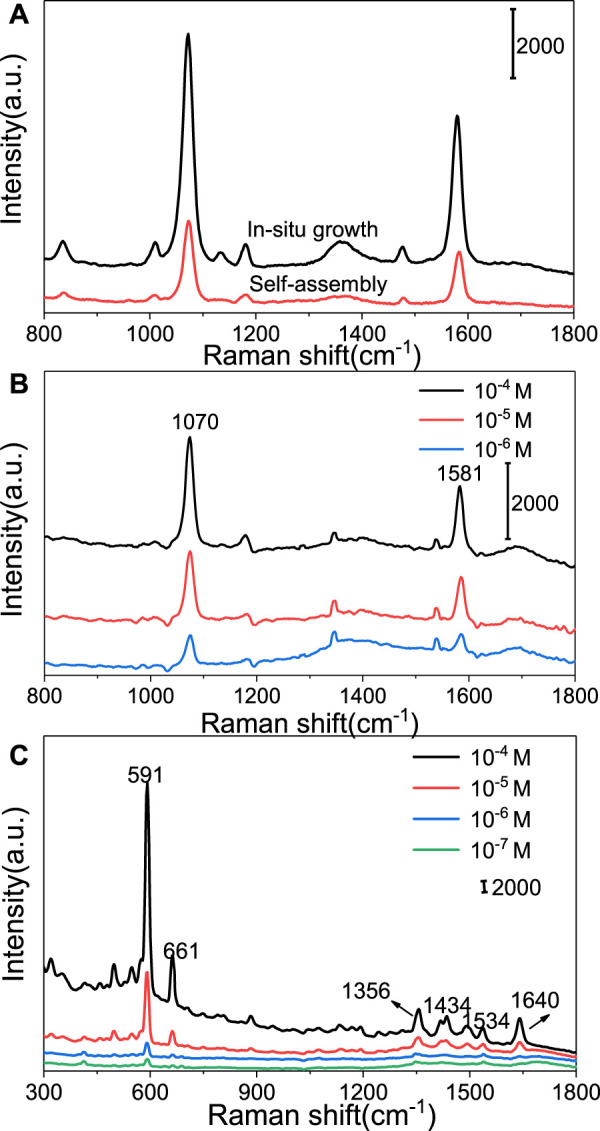
Surface-enhanced Raman scattering (SERS) spectra of MBA from Ag@PS *via in situ* growth and self-assembled methods **(A)**. SERS spectra of different concentrations of MBA **(B)** and Nile blue A (NBA) **(C)** on Ag@PS composite.

The SERS activity of the plasmonic Ag@PS prepared by the *in situ* growth strategy was compared with that constructed through the self-assembly process. Both plasmonic composites were mixed with MBA at 0.1 mM, and the Raman spectra were measured under the same conditions. As presented in Figure 6A, the Ag@PS composite, prepared through the *in situ* growth method, exhibits more intense SERS intensity than that prepared by the self-assemble method. The density of Ag on the Ag@PS composite prepared by the self-assemble method was low as shown in Supplementary Figure S2. The obvious enhancement effect mainly arose from the densely packed Ag from Ag@PS prepared *via* the *in situ* growth method.

The SERS activity of the Ag@PS composite was evaluated by using 4-MBA and NBA as a probe molecule. Figure 6B shows the SERS spectra of 4-MBA at different concentrations (10^−4^–10^–6^ M). The prominent Raman bands of MBA were still obtained even when the concentration went down to 10^–6^ M. NBA was also selected as an analyte for evaluating SERS enhancement of Ag@PS. The Ag@PS composites were mixed with the aqueous solution of NBA at different concentrations. Figure 6C shows the SERS signal of NBA measured from Ag@PS. The characteristic Raman peaks of NBA on Ag@PS composites were mainly located at 591, 661, 1,356, 1,434, 1,534, and 1,640 cm^−1^. The intensity of SERS signal of NBA was monotonously decreased as the NBA concentration decreased. When the concentration of NBA was down to 10^–7^ M, the characteristic Raman peak of NBA at 591 cm^−1^ was still observed. Thus, the Ag@PS composite, prepared via the *in situ* growth method, was very active and promising for use in SERS sensing.

**FIGURE 7:**
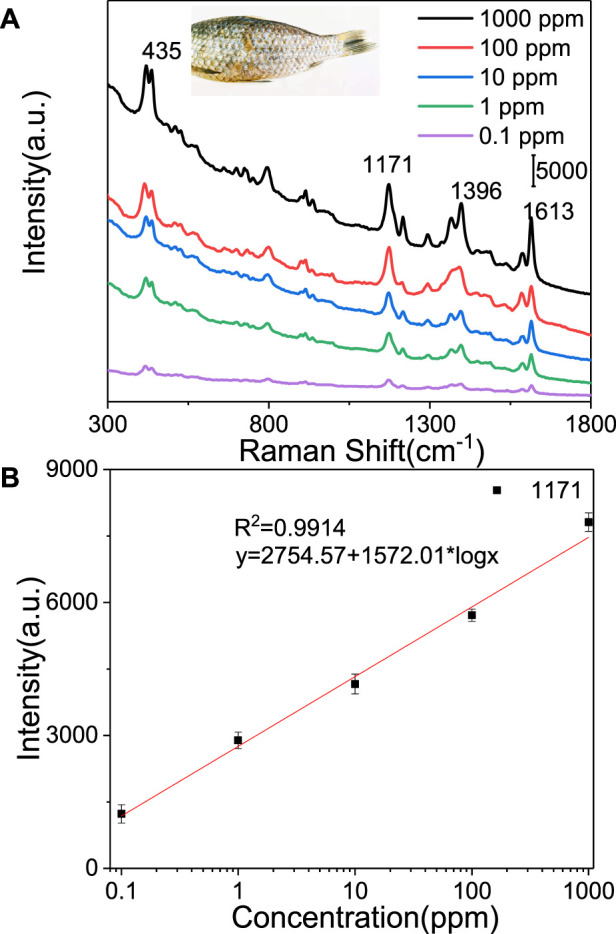
**(A)** SERS spectra of MG from fish using the Ag@PS substrate. **(B)** SERS intensity at 1,171 cm^−1^ vs. logarithm of the concentrations of MG.

The Ag@PS composite was used to detect MG from fish. Five microliters of Ag@PS (4 mg/ml) was dropped onto the surface of fish with different concentrations of MG, and after 3 min, the Raman signal was collected and is presented in Figure 7A. Several Raman peaks were observed at 435, 1,171, 1,396, and 1,613 cm^−1^. The prominent band at 435 cm^−1^ was assigned to the vibration of phenyl-C-phenyl. The peak at 1,613 cm^−1^ was associated with the stretching vibration of C–C bond in the aromatic ring (Yuanyi Zhang et al., 2021). The intensity of Raman peaks was decreased as the concentration of MG decreased. The characteristic peaks of MG at 1,171 and 1,613 cm^−1^ still need to be measured as the concentration of MG went down to 0.1 ppm. The intensity of the Raman band at 1,171 cm^−1^ was chosen in establishing a relationship with the concentration of MG; the liner relationship curve is presented in Figure 7B. These results indicate that Ag@PS could be used as a fast, simple, and convenient SERS platform in detecting MG.

**FIGURE 8:**
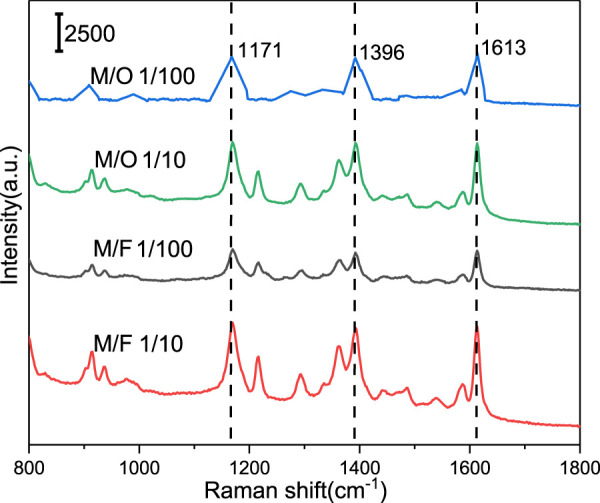
Detection of MG from different mixtures by Ag@PS SERS substrate.

The mixed pesticides usually used for protecting fish from disease in aquaculture, and several kinds of pesticides would exist in the fish. Oxytetracycline and furazolidone were commonly used in the aquaculture and agriculture. Therefore, four mixtures composed of MG/oxytetracycline and MG/furazolidone at different ratios (M/O, 1/10; M/O, 1/100; M/F, 1/10; M/F, 1/100) were used as target analytes. Figure 8 presents the SERS spectra of four mixtures measured from Ag@PS, in which the feature Raman peaks of MG were observed. The results indicated that the Ag@PS SERS composite has excellent selectivity to MG in SERS sensing.

## Conclusion

A simple, rapid, and efficient method was developed to prepare the Ag@PS composite. The high density of Ag NPs was decorated onto the PS microspheres *via* a seed-mediated *in situ* growth process. The SERS enhancement could be controlled by adjusting the concentration of AgNO_3_ and AA in the growth medium. When the concentration of AgNO_3_ and AA were 20 and 30 mM, respectively, the Ag@PS composite showed the best SERS enhancement and thermal stability. Ag@PS was used as a SERS substrate to detect 0.1 ppm of MG on the surface of fish with excellent selectivity. The Ag@PS composite shows a potential application in food safety and environment monitoring.

## Data Availability

The raw data supporting the conclusion of this article will be made available by the authors, without undue reservation.

## References

[B1] Balčytis A. Ryu M. Seniutinas G. Stoddart P. R. Al Mamun M. A. Morikawa J. (2017). Nano-rescaling of Gold Films on Polystyrene: thermal Management for SERS. Nanoscale 9 (2), 690–695. 10.1039/C6NR06904E 27957572

[B2] Bergwerff A. A. Scherpenisse P. (2003). Determination of Residues of Malachite green in Aquatic Animals. J. Chromatogr. B 788 (2), 351–359. 10.1016/S1570-0232(03)00042-4 12705975

[B3] Cai W. Wang W. Lu L. Chen T. (2013). Coating Sulfonated Polystyrene Microspheres with Highly Dense Gold Nanoparticle Shell for SERS Application. Colloid Polym. Sci. 291 (8), 2023–2029. 10.1007/s00396-013-2928-7

[B4] Cao Y. C. Jin R. Mirkin C. A. (2002). Nanoparticles with Raman Spectroscopic Fingerprints for DNA and RNA Detection. Science 297 (5586), 1536–1540. 10.1126/science.297.5586.1536 12202825

[B5] Cao Y.-C. Wang Z. Jin X. Hua X.-F. Liu M.-X. Zhao Y.-D. (2009). Preparation of Au Nanoparticles-Coated Polystyrene Beads and its Application in Protein Immobilization. Colloids Surf. A: Physicochemical Eng. Aspects 334 (1-3), 53–58. 10.1016/j.colsurfa.2008.10.002

[B7] Chang C.-P. Tseng C.-C. Ou J.-L. Hwu W.-H. Ger M.-D. (2010). Growth Mechanism of Gold Nanoparticles Decorated on Polystyrene Spheres via Self-Regulated Reduction. Colloid Polym. Sci. 288 (4), 395–403. 10.1007/s00396-009-2134-9

[B8] El-Khiyami S. S. Ismail A. M. Hafez R. S. (2021). Characterization, Optical and Conductivity Study of Nickel Oxide Based Nanocomposites of Polystyrene. J. Inorg. Organomet. Polym. 31, 4313–4325. 10.1007/s10904-021-02041-x

[B9] Fleischmann M. Hendra P. J. McQuillan A. J. (1974). Raman Spectra of Pyridine Adsorbed at a Silver Electrode. Chem. Phys. Lett. 26 (2), 163–166. 10.1016/0009-2614(74)85388-1

[B10] Forgacs E. Cserháti T. Oros G. (2004). Removal of Synthetic Dyes from Wastewaters: a Review. Environ. Int. 30 (7), 953–971. 10.1016/j.envint.2004.02.001 15196844

[B43] Gao T. Wang Y. Wang K. Zhang X. Dui J. Li G. (2013). Controlled Synthesis of Homogeneous Ag Nanosheet-Assembled Film for Effective SERS Substrate. ACS Appl. Mater. Inter. 5 (15), 7308–7314. 10.1021/am401552x 23829572

[B11] Gittins D. I. Susha A. S. Schoeler B. Caruso F. (2002). Dense Nanoparticulate Thin Films via Gold Nanoparticle Self-Assembly. Adv. Mater. 14 (7), 508–512. 10.1002/1521-4095(20020404)14:7<508::aid-adma508>3.0.co;2-t

[B20] Gong J. Zu X. Mu W. Deng Y. (2013). *In Situ* self-assembly Synthesis of Gold Nanoparticle Arrays on Polystyrene Microspheres and Their Surface Plasmon Resonance. Colloid Polym. Sci. 291 (1), 239–244. 10.1007/s00396-012-2601-6

[B12] Gong Z. Du H. Cheng F. Wang C. Wang C. Fan M. (2014). Fabrication of SERS Swab for Direct Detection of Trace Explosives in Fingerprints. ACS Appl. Mater. Inter. 6 (24), 21931–21937. 10.1021/am507424v 25455731

[B13] Halme K. Lindfors E. Peltonen K. (2007). A Confirmatory Analysis of Malachite green Residues in Rainbow trout with Liquid Chromatography-Electrospray Tandem Mass Spectrometry. J. Chromatogr. B 845 (1), 74–79. 10.1016/j.jchromb.2006.07.048 16931188

[B14] Haruna K. Saleh T. A. Hossain M. K. Al-Saadi A. A. (2016). Hydroxylamine Reduced Silver Colloid for Naphthalene and Phenanthrene Detection Using Surface-Enhanced Raman Spectroscopy. Chem. Eng. J. 304, 141–148. 10.1016/j.cej.2016.06.050

[B15] He L. Kim N.-J. Li H. Hu Z. Lin M. (2008). Use of a Fractal-like Gold Nanostructure in Surface-Enhanced Raman Spectroscopy for Detection of Selected Food Contaminants. J. Agric. Food Chem. 56 (21), 9843–9847. 10.1021/jf801969v 18828599

[B16] Heras H. Pollero R. J. (1990). New Phase Separator for Extraction-Spectrophotometric Determination of Anionic Surfactants with Malachite Green by Flow Injection Analysis. J. Exp. Mar. Biol. Ecol. 140 (1-2), 29–38. 10.1016/S0039-9140(97)00096-910.1016/0022-0981(90)90078-q 18967035

[B17] Hou H. Wang P. Zhang J. Li C. Jin Y. (2015). Graphene Oxide-Supported Ag Nanoplates as LSPR Tunable and Reproducible Substrates for SERS Applications with Optimized Sensitivity. ACS Appl. Mater. Inter. 7 (32), 18038–18045. 10.1021/acsami.5b04946 26203672

[B18] Hu X. Xu Z. Li K. Fang F. Wang L. (2015). Fabrication of a Au-Polystyrene Sphere Substrate with Three-Dimensional Nanofeatures for Surface-Enhanced Raman Spectroscopy. Appl. Surf. Sci. 355 (NOV.15), 1168–1174. 10.1016/j.apsusc.2015.07.215

[B19] Ishida T. Kuroda K. Kinoshita N. Minagawa W. Haruta M. (2008). Direct Deposition of Gold Nanoparticles onto Polymer Beads and Glucose Oxidation with H2O2. J. Colloid Interf. Sci. 323 (1), 105–111. 10.1016/j.jcis.2008.02.046 18387617

[B21] Jin E. Guo J. Yang F. Zhu Y. Song J. Jin Y. (2016). On the Polymorphic and Morphological Changes of Cellulose Nanocrystals (CNC-I) upon Mercerization and Conversion to CNC-II. Carbohydr. Polym. 143, 327–335. 10.1016/j.carbpol.2016.01.048 27083376

[B22] Kamińska A. Witkowska E. Kowalska A. Skoczyńska A. Gawryszewska I. Guziewicz E. (2016). Highly Efficient SERS-Based Detection of Cerebrospinal Fluid Neopterin as a Diagnostic Marker of Bacterial Infection. Anal. Bioanal. Chem. 408 (16), 4319–4327. 10.1007/s00216-016-9553-710.1007/s00216-016-9535-7 27086021 PMC4875960

[B23] Kim Y.-K. Min D.-H. (2014). Surface Confined Successive Growth of Silver Nanoplates on a Solid Substrate with Tunable Surface Plasmon Resonance. RSC Adv. 4 (14), 6950–6956. 10.1039/C3RA44280B

[B24] Kim H. Daniels E. S. Dimonie V. L. Klein A. (2008). Nucleation of Gold Nanoparticles on Latex Particle Surfaces. J. Polym. Sci. A. Polym. Chem. 46 (3), 912–925. 10.1002/pola.22434

[B25] Kneipp J. Kneipp H. Kneipp K. (2008). SERS-a Single-Molecule and Nanoscale Tool for Bioanalytics. Chem. Soc. Rev. 37 (5), 1052–1060. 10.1039/B708459P 18443689

[B26] Kong X.-M. Reza M. Ma Y.-B. Hinestroza J.-P. Ahvenniemi E. Vuorinen T. (2015). Assembly of Metal Nanoparticles on Regenerated Fibers from wood Sawdust and De-inked Pulp: Flexible Substrates for Surface Enhanced Raman Scattering (SERS) Applications. Cellulose 22 (6), 3645–3655. 10.1007/s10570-015-0743-7

[B27] Kong X. Chong X. Squire K. Wang A. X. (2018). Microfluidic Diatomite Analytical Devices for Illicit Drug Sensing with Ppb-Level Sensitivity. Sensors Actuators B: Chem. 259, 587–595. 10.1016/j.snb.2017.12.038 PMC5943051 29755211

[B28] Lee J.-H. Kim D. O. Song G.-S. Lee Y. Jung S.-B. Nam J.-D. (2007). Direct Metallization of Gold Nanoparticles on a Polystyrene Bead Surface Using Cationic Gold Ligands. Macromol. Rapid Commun. 28 (5), 634–640. 10.1002/marc.200600757

[B29] Li Z. Meng G. Liang T. Zhang Z. Zhu X. (2013). Facile Synthesis of Large-Scale Ag Nanosheet-Assembled Films with Sub-10nm Gaps as Highly Active and Homogeneous SERS Substrates. Appl. Surf. Sci. 264, 383–390. 10.1016/j.apsusc.2012.10.031

[B30] Li P. Dong R. Wu Y. Liu H. Kong L. Yang L. (2014). Polystyrene/Ag Nanoparticles as Dynamic Surface-Enhanced Raman Spectroscopy Substrates for Sensitive Detection of Organophosphorus Pesticides. Talanta 127, 269–275. 10.1016/j.talanta.2014.03.075 24913887

[B47] Li Y. Ye Y. Fan Y. Zhou J. Jia L. Tang B. (2017). Silver Nanoprism-Loaded Eggshell Membrane: A Facile Platform for *In Situ* SERS Monitoring of Catalytic Reactions. Crystals 7 (2), 45. 10.3990/cryst702004510.3390/cryst7020045

[B31] Lin X. Fang G. Liu Y. He Y. Wang L. Dong B. (2020). Marangoni Effect-Driven Transfer and Compression at Three-phase Interfaces for Highly Reproducible Nanoparticle Monolayers. J. Phys. Chem. Lett. 11 (9), 3573–3581. 10.1021/acs.jpclett.0c01116 32293181

[B44] Liu W. Yang X. Xie L. (2007). Size-controlled Gold Nanocolloids on Polymer Microsphere-Stabilizer via Interaction between Functional Groups and Gold Nanocolloids. J. Colloid Interf. Sci. 313 (2), 494–502. 10.1016/j.jcis.2007.04.055 17540399

[B6] Lu L. Randjelovic I. Capek R. Gaponik N. Yang J. Zhang H. (2005). Controlled Fabrication of Gold-Coated 3D Ordered Colloidal Crystal Films and Their Application in Surface-Enhanced Raman Spectroscopy. Chem. Mater. 17 (23), 5731–5736. 10.1021/cm051473d

[B32] Marquette C. A. Hezard P. Degiuli A. Blum L. J. (2006). Macro-molecular Chemiluminescent Complex for Enhanced Immuno-Detection onto Microtiter Plate and Protein Biochip. Sensors Actuators B: Chem. 113 (2), 664–670. 10.1016/j.snb.2005.07.015

[B33] Oćwieja M. Lupa D. Adamczyk Z. (2018). Gold Nanoparticle Layers on Polystyrene Microspheres of Controlled Structure and Electrokinetic Properties. Langmuir 34 (29), 8489–8498. 10.1021/acs.langmuir.8b01491 29936835

[B34] Onawole A. T. Popoola S. A. Saleh T. A. Al-Saadi A. A. (2018). Silver-loaded Graphene as an Effective SERS Substrate for Clotrimazole Detection: DFT and Spectroscopic Studies. Spectrochimica Acta A: Mol. Biomol. Spectrosc. 201, 354–361. 10.1016/j.saa.2018.05.018 29763829

[B35] Robinson A. M. Zhao L. Shah Alam M. Y. Bhandari P. Harroun S. G. Dendukuri D. (2015). The Development of "Fab-Chips" as Low-Cost, Sensitive Surface-Enhanced Raman Spectroscopy (SERS) Substrates for Analytical Applications. Analyst 140 (3), 779–785. 10.1039/C4AN01633E 25460852

[B37] Sivashanmugan K. Liao J.-D. Liu B. H. Yao C.-K. Luo S.-C. (2015). Ag Nanoclusters on ZnO Nanodome Array as Hybrid SERS-Active Substrate for Trace Detection of Malachite green. Sensors Actuators B: Chem. 207, 430–436. 10.1016/j.snb.2014.10.088

[B38] Sivashanmugan K. Squire K. Kraai J. A. Tan A. Zhao Y. Rorrer G. L. (2019a). Biological Photonic Crystal‐Enhanced Plasmonic Mesocapsules: Approaching Single‐Molecule Optofluidic‐SERS Sensing. Adv. Opt. Mater. 7 (13), 1900415. 10.1002/adom.201900415 32775144 PMC7410161

[B39] Sivashanmugan K. Squire K. Tan A. Zhao Y. Kraai J. A. Rorrer G. L. (2019b). Trace Detection of Tetrahydrocannabinol in Body Fluid via Surface-Enhanced Raman Scattering and Principal Component Analysis. ACS Sens. 4 (4), 1109–1117. 10.1021/acssensors.9b00476 30907578

[B40] Stamplecoskie K. G. Scaiano J. C. Tiwari V. S. Anis H. (2011). Optimal Size of Silver Nanoparticles for Surface-Enhanced Raman Spectroscopy. J. Phys. Chem. C 115 (5), 1403–1409. 10.1021/jp106666t

[B41] Stead S. L. Stead H. A. Ashwin H. Johnston B. H. Dallas A. Kazakov S. A. (2010). Anne Dallas, SergeiAn RNA-Aptamer-Based Assay for the Detection and Analysis of Malachite green and Leucomalachite green Residues in Fish Tissue. Anal. Chem. 82 (7), 2652–2660. 10.1021/ac902226v 20201504

[B42] Tao A. Kim F. Hess C. Goldberger J. He R. Sun Y. (2003). Langmuir−Blodgett Silver Nanowire Monolayers for Molecular Sensing Using Surface-Enhanced Raman Spectroscopy. Nano Lett. 3 (9), 1229–1233. 10.1021/nl0344209

[B45] Xiao M. Chen H. Ming T. Shao L. Wang J. (2010). Plasmon-modulated Light Scattering from Gold Nanocrystal-Decorated Hollow Mesoporous Silica Microspheres. Acs Nano 4 (11), 6565–6572. 10.1021/nn101804v 20939510

[B46] Xu T. Wang X. Huang Y. Lai K. Fan Y. (2019). Rapid Detection of Trace Methylene Blue and Malachite green in Four Fish Tissues by Ultra-sensitive Surface-Enhanced Raman Spectroscopy Coated with Gold Nanorods. Food Control 106, 106720. 10.1016/j.foodcont.2019.106720

[B48] Yang M.-C. Fang J.-M. Kuo T.-F. Wang D.-M. Huang Y.-L. Liu L.-Y. (2007). Production of Antibodies for Selective Detection of Malachite green and the Related Triphenylmethane Dyes in Fish and Fishpond Water. J. Agric. Food Chem. 55 (22), 8851–8856. 10.1021/jf071195y 17924699

[B49] Zhao Y. Li X. Liu Y. Zhang L. Wang F. Lu Y. (2017). High Performance Surface-Enhanced Raman Scattering Sensing Based on Au Nanoparticle-Monolayer Graphene-Ag Nanostar Array Hybrid System. Sensors Actuators B: Chem. 247, 850–857. 10.1016/j.snb.2017.03.063

[B50] Zhang Y. Huang Y. Kang Y. Miao J. Lai K. (2021). Selective Recognition and Determination of Malachite green in Fish Muscles via Surface-Enhanced Raman Scattering Coupled with Molecularly Imprinted Polymers. Food Control 130 (1–2), 108367. 10.1016/j.foodcont.2021.108367

[B51] Zhang Y. Lai K. Zhou J. Wang X. Rasco B. A. Huang Y. (2012). A Novel Approach to Determine Leucomalachite green and Malachite green in Fish Fillets with Surface-Enhanced Raman Spectroscopy (SERS) and Multivariate Analyses. J. Raman Spectrosc. 43, 1208–1213. 10.1002/jrs.4050

[B52] Zhang H. liu M. Zhou F. Liu D. Liu G. Duan G. (2015). Physical Deposition Improved SERS Stability of Morphology Controlled Periodic Micro/Nanostructured Arrays Based on Colloidal Templates. Small 11 (7), 844–853. 10.1002/smll.201402630 25356821

[B53] Zhang C. You T. Yang N. Gao Y. Jiang L. Yin P. (2019). Hydrophobic Paper-Based SERS Platform for Direct-Droplet Quantitative Determination of Melamine. Food Chem. 287, 363–368. 10.1016/j.foodchem.2019.02.094 30857711

[B36] Zhang S. Xu Z. Guo J. Wang H. Ma Y. Kong X. (2021). Layer-by-Layer Assembly of Polystyrene/Ag for a Highly Reproducible SERS Substrate and its Use for the Detection of Food Contaminants. Polymers 13 (19), 3270. 10.3390/polym13193270 34641085 PMC8512144

