# Supplementary material for: Preparation of Plasmonic Ag@PS Composite via Seed-Mediated In Situ Growth Method and Application in SERS
Source: Front Chem. 2022 Mar 11;10:847203. doi: 10.3389/fchem.2022.847203 (PMC8963369; doi:10.3389/fchem.2022.847203)
Supplement: Supplementary file 1 [file DataSheet1.docx]

**Preparation of plasmonic PS@Ag composite via seed-mediated in-situ growth method and application in SERS**

Xiaoran Tian^a^, Qian Yu^a^, Xianming Kong^a*^ Miao Zhang ^b*^

^a^ School of Petrochemical Engineering, Liaoning Petrochemical University, Fushun, Liaoning 113001, P. R. China

^b^ Department of Materials and Environmental Chemistry, Stockholm University, 10691 Stockholm, Sweden

*Corresponding author: xmkong@lnpu.edu.cn; miao.zhang @mmk.su.se


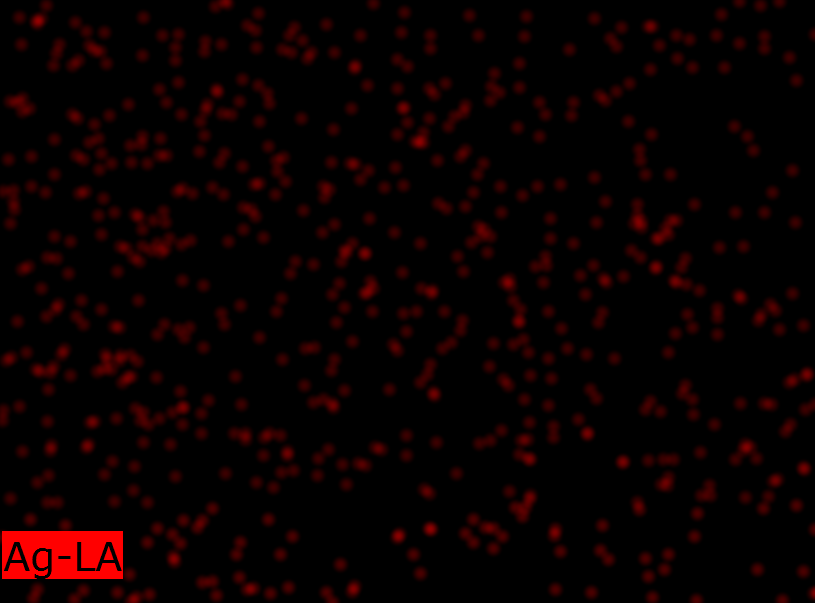


Fig.S1. Elemental mapping image of Ag on Ag@PS composite


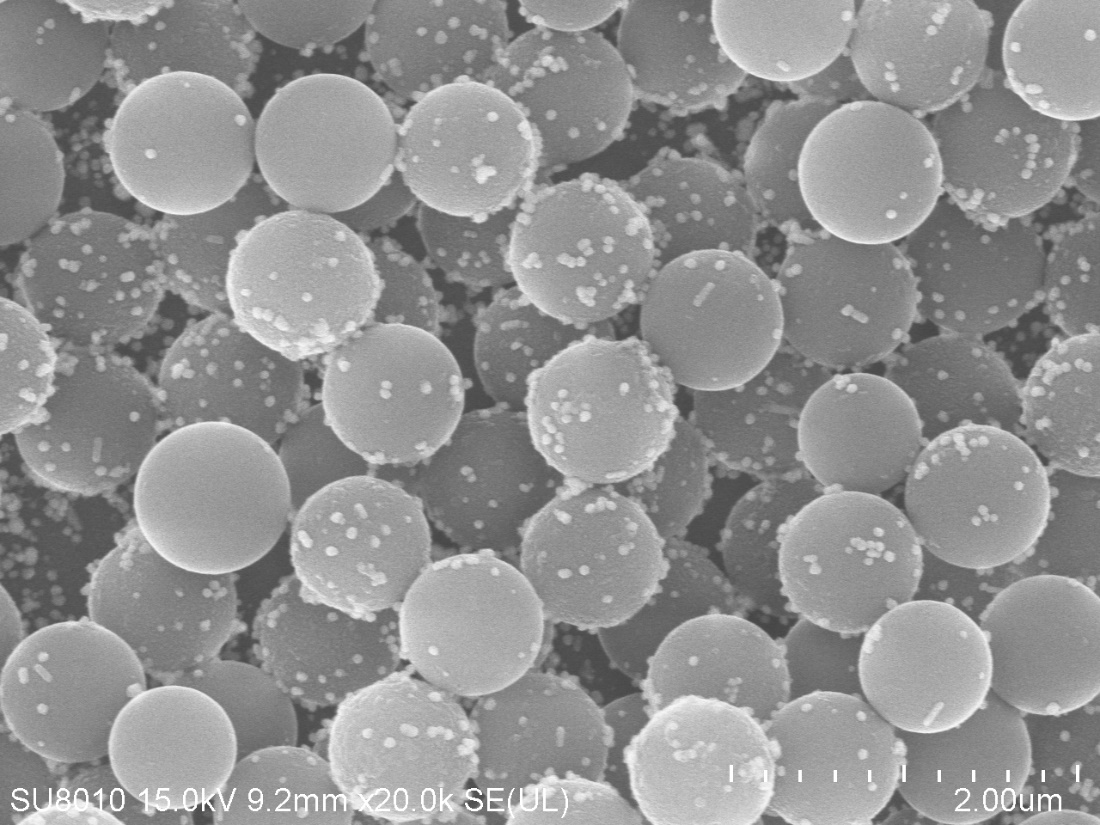


Fig.S2. SEM image of PS/Ag fabricated by self-assemble method
